# High Prevalence of Human Papillomavirus in Colorectal Cancer in Hispanics: A Case-Control Study

**DOI:** 10.1155/2016/7896716

**Published:** 2016-01-19

**Authors:** Raul D. Bernabe-Dones, Maria Gonzalez-Pons, Alejandro Villar-Prados, Mercedes Lacourt-Ventura, Heriberto Rodríguez-Arroyo, Sharon Fonseca-Williams, Francisco E. Velazquez, Yaritza Diaz-Algorri, Sofia M. Lopez-Diaz, Nayra Rodríguez, Yasuhiro Yamamura, Marcia Cruz-Correa

**Affiliations:** ^1^School of Health Professions, University of Puerto Rico Medical Sciences Campus, San Juan, PR 00935, USA; ^2^Division of Gastrointestinal Oncology, University of Puerto Rico Comprehensive Cancer Center, San Juan, PR 00935, USA; ^3^AIDS Research Program, Ponce Health Sciences University School of Medicine, Ponce, PR, USA; ^4^Department of Surgery, Biochemistry and Medicine, University of Puerto Rico Medical Sciences Campus, San Juan, PR 00716, USA

## Abstract

The role of Human Papillomavirus (HPV) in colorectal carcinogenesis remains elusive. Based on the high incidence of HPV-associated malignancies among Puerto Rican Hispanics, this study aimed to assess the prevalence of HPV infection and viral integration in colorectal tissues in order to evaluate its putative role in colorectal cancer (CRC). In this case-control study, the prevalence of HPV infection in CRC (cases *n* = 45) and normal colon mucosa from cancer-free subjects (controls *n* = 36) was assessed by a nested PCR strategy. HPV-16 genotyping was performed in HPV-positive tissues and the physical status of the HPV-16 genome was determined by E2 detection. HPV was detected in 19 of 45 (42.2%) CRC cases (mean age 61.1 ± 10.7 years, 24 males) and in 1 of 36 (2.8%) controls (mean age 60.9 ± 9.6 years, 24 males) with an OR = 25.58 (95% CI 3.21 to 203.49). HPV-16 was detected in 63.2% of the HPV-positive colorectal tumors; genome integration was observed in all HPV-16 positive cases. This is the first report showing the high prevalence of HPV infections in Caribbean Hispanic colorectal tumors. Despite evidence of HPV integration into the host genome, further mechanistic analysis examining HPV oncoprotein expression and the putative role of these oncoproteins in colorectal carcinogenesis is warranted.

## 1. Introduction

Human Papillomavirus (HPV) infections are the most common sexually transmitted infections in the United States (US) (http://www.cdc.gov/std/hpv/STDFact-HPV.htm). HPVs are epitheliotropic, double-stranded DNA viruses that infect the squamous epithelia of mucosal cells in the skin. Viral multiplication occurs in the cell nuclei and is tightly linked to the cell's differentiation state. There are well over 100 genotypes of HPV. Types 6, 11, 40, and 42 are commonly associated with benign lesions and are classified as low-risk types. HPV-16, HPV-18, HPV-31, and HPV-45 are considered to possess high oncogenic potential and are referred to as high-risk types [1, 2]. HPV has been identified as a causal agent in cervical [3–5], vaginal [6], anal [7], oral [8], and penile cancers [9]. Studies have also shown strong correlations between HPV and the development of many types of cancers such as esophagus [10], pharynx [11], and larynx [12]. However, the putative role of HPV infection in colorectal carcinogenesis has not been properly elucidated and still remains controversial.

The pathogenesis of CRC has become better understood at the molecular level; however, the etiology of CRC is still incompletely understood. In the last decade, several studies have suggested that HPV might have a role in colorectal carcinogenesis [13, 14]. Detection of HPV in colonic tissue has led to a wide range of work proposing a causal association between HPV and CRC. The presence of HPV in colon tissue remains a highly controversial topic of discussion because of inconsistencies in result reproducibility. Since integration of the HPV genome is necessary for the virus to exert its carcinogenic potential, assessment of the physical status of the viral genome after confirming HPV infection is essential to establish a causal association. Inactivation of the E2 gene through genomic integration promotes the expression of the E6 and E7 oncoproteins, which antagonize the function of p53 and pRB, respectively [4, 15]. The resulting degradation of p53 and pRB mediated by these oncoproteins facilitates viral DNA proliferation within the host and leads to neoplasia by mechanisms well described throughout the literature [16, 17].

CRC is responsible for approximately 694,000 deaths worldwide [18]. In the USA, CRC is the 3rd most commonly diagnosed cancer and the 3rd leading cause of cancer death [19]. In Puerto Rico (PR), CRC is the 2nd most diagnosed and the leading cause of cancer death among men and women [20]. A high prevalence of HPV-related cancers [21–23] and a high prevalence of HPV in anogenital samples have been reported among Puerto Rican men and women [24]. The seroprevalence of HPV-16 was reported to be 11.3% in a population-based sample of adults in PR [25], which is similar to that reported in the USA (11.5%) [26]. Thus, given the high CRC mortality rates and the high incidence of HPV related morbidities in PR, the overall aim of this case-control study was to evaluate the association between CRC and HPV infection in samples from Puerto Rican Hispanics.

## 2. Methods

### 2.1. Ethics Statement

This study was approved by the University of Puerto Rico Medical Sciences Campus IRB (#A7330109). All procedures were in accordance with the ethical standards of the IRB.

### 2.2. Subject Recruitment and Sample Acquisition

Cases and controls were recruited consecutively using convenience sampling and were frequency-matched by gender and age. Study participants aged 21 years or older were recruited when they visited the Puerto Rico Medical Center facilities for colonoscopies due to routine screening, symptoms, and/or referrals by gastroenterologists and colorectal surgeons. The purpose of this study was explained and all study participants gave informed consent prior to inclusion. All of the participants included in this analysis were Hispanics. Being Hispanic was defined by the participant's self-reported heritage or place of birth. All of the control subjects included in this study had normal results in their colonoscopy. Individuals with CRC had a diagnosis of adenocarcinoma confirmed by histopathology. Subjects with hereditary cancer syndromes and inflammatory bowel disease were excluded.

Fresh frozen tissue was obtained from 45 nonfamilial, sporadic CRC patients (cases) and 36 cancer-free individuals (controls). Tissues were harvested from CRC patients during tumor resection surgery. Anal or perianal tumors and tissue samples from individuals who reported to be HIV-positive were excluded. Tumor location was classified as* proximal* (from the cecum to the distal transverse colon),* distal* (from the splenic flexure to the sigmoid colon), or* rectum* (last 20 cm of the colon). Control colorectal tissue biopsies were obtained from the distal colon during routine colonoscopies.

Using the Collaborative Family Registries' Colorectal Cancer Risk Factor Questionnaire (http://coloncfr.org/), clinical and sociodemographic data was collected from each patient including gender, lifestyle, medical history, and family history of cancer. The sociodemographic and clinical characteristics analyzed in the study were gender (male versus female), median age (<61 years versus ≥61 years), type 2 DM diagnosis (yes versus no), family history of any cancer (yes versus no), and family history of CRC (yes versus no). Family history is defined as having a first-, second-, or third-degree relative with cancer. The lifestyle characteristics that were analyzed in this study were alcohol consumption (ever versus never) and smoking status (ever versus never).

### 2.3. DNA Extraction

Genomic DNA (gDNA) was extracted from ≈100 mg of tissue using the DNA Isolation Kit for Cells and Tissues (Roche Applied Science, Indianapolis, IN) following the manufacturer's instructions. For quality control purposes, *β-actin* gene PCR amplification was used to assess gDNA integrity; samples from which *β-actin* could not be amplified were excluded from the study.

The following standardized precautions were taken to minimize sample-to-sample cross-contamination: all instruments and work benches were wiped down with DNAZap (Ambion, Foster City, CA), followed by 10% bleach and 70% ETOH prior to sample manipulation. New, sterile blades were used for each sample. Tissue processing and nucleotide extraction were limited to a maximum of 10 samples per day. Approximately 100 mg of each specimen was randomly coded in a blinded manner and used for further analyses.

### 2.4. HPV Detection and Genotyping

Human HPV DNA detection was performed via a nested PCR strategy using PGMY09/PGMY11 as outer primers and GP5+/GP6+ as inner primers [27, 28]. These primers amplify an HPV L1 consensus region that detects more than 25 HPV types. Each reaction was carried out in a total volume of 25 *μ*L containing 200 ng of gDNA, 12.5 *μ*L of Bullseye HS-Taq 2x Master Mix (MidSci, St. Louis, MO), and 100 nmol/L of pooled PGMY09/11 primers. The following conditions were used: 15 minutes at 95°C, followed by 35 cycles of 60 seconds each at 94°C, 60°C, and 72°C, with a final extension of 10 minutes at 72°C. Two microliters of the first PCR reaction was used as the template for the nested PCR. The conditions for the nested PCR were identical to the first run PCR with the exception of the annealing temperature which was 52°C. Amplified products were electrophoresed and analyzed using a ChemiDoc (Bio-Rad, Hercules, CA). Detection of pHPV-16 purified plasmid DNA (ATCC 45113D) was used as a positive control and water as a negative control.

HPV-16 DNA genotyping was carried out with the type-specific primers targeting HPV-16 E6 [13]. HPV-16E6 Pr80 (5′-CTGACTCGAG/TTTATGCACCAAAAGAGAAC-3′) and Pr625 (5′-GATCAGTTGTCTCTGGTTGC-3′) primers were used for the first run, with the same PCR conditions described above with the exception of the annealing temperature which was 68°C. The conditions for the nested PCR with primers Pr106: 5′-GTTTCAGGACCCACAGGAGC-3′ and Pr562: 5′-GTACTCACC CC/TGATTACAGCTGGGTTT C-3′ were the same with the exception of the annealing temperature which was 60°C. Detection of the pHPV-16 purified plasmid DNA (ATCC 45113D) and water were used as positive and negative controls, respectively. Amplicons were electrophoresed and visualized as described. Ten percent of the samples tested by nested PCR were externally validated and confirmed at the AIDS Research Program Laboratory at the Ponce School of Medicine & Health Sciences (Ponce, PR) using the INNO-LiPA HPV Genotyping Extra kit (Fujirebio, Gent, Belgium).

### 2.5. HPV Physical Status

The physical status of the HPV-16 genome was determined by examining E2 sequence integrity using a previously described nested PCR strategy [29]. All tumors harboring HPV-16 DNA were analyzed (*n* = 12). Nested PCR analysis was performed using 2 sets of specific HPV-16 primers which included 2 forward primers, Pr7581 (5′-CACTGCTTGCCAACCATTCC-3′) and Pr7677 (5′-GCC AAC GCC TTA CAT ACC G-3′), and 2 reverse primers, Pr128 (5′-GTCGCTCCTGTGGGTCCTG-3′) and Pr223 (5′-ACGTCGCAGTAACTGTTGC-3′). Both PCRs were performed under the same conditions previously described with the exception of the annealing temperature which was 60°C. Amplicons were analyzed as described. DNA extracted from CaSki (CRL-1550) and SiHa (HTB-35) human cervical cell lines were used as positive controls. The absence of PCR product was interpreted as E2 sequence disruption and integration of viral DNA into the host genome. The presence of E2 amplicons indicates the presence of episomal HPV-16 genomes.

### 2.6. Statistical Analysis

Sociodemographic, clinical, and lifestyle characteristics in cases and controls were described using frequency distributions for categorical variables and summary measures for quantitative variables. Two-sided tests were used to compare study groups: the chi-square test or Fisher's exact test was used for categorical variables and Student's *t*-test or Mann-Whitney test to compare quantitative variables. Unconditional logistic regression models were used to estimate the OR with 95% confidence of CRC in relation to HPV status and other variables. Statistical analyses were performed using SPSS 17.0 (SPSS Inc.) and Epi InfoTM 7 (CDC).

## 3. Results

### 3.1. Sociodemographic, Lifestyle, and Clinical History Characteristics of Study Participants

The demographic and clinical characteristics of the study population (*n* = 81; 45 CRC cases and 36 controls) are presented in Table 1. The mean age of the CRC cases was 61.1 years (ranging from 38 to 86 years; 24 were males). In the control group, the mean age was 60.9 years (ranging from 42 to 85 years; 15 were males). Compared to controls, CRC cases were more likely to report a family history of CRC (OR 0.29; 95% 0.10–0.80). In terms of the ages of the family members with CRC as given by the subject, the median age of CRC diagnosis in the relatives of cases and controls was 60 years. No statistically significant associations were found when comparing cases and controls with relatives diagnosed with CRC at ≥60 years versus <60 years. No other significant associations were found.

### 3.2. HPV DNA Detection

**Figure 1:**
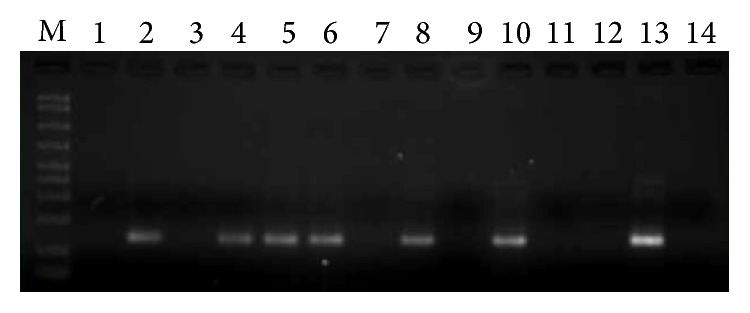
HPV detection assay. This figure shows the electrophotetic profile of 12 representative cases assayed by nested PCR for the detection of* HPV L1*. Cases 2, 4, 5, 6, 8, and 10 are positive as demonstrated by the presence of the amplicon generated by the GP 5+/6+ primer pair. Lane M is the molecular size marker; lanes 13 and 14 were HPV positive and water controls, respectively.

A significant difference in the prevalence of HPV infection was observed when comparing colorectal tumor tissues and normal mucosa from controls. HPV DNA was detected in 19 of the 45 (42.2%) CRC samples and in 1 of the 36 (2.8%) control samples studied (Figure 1). HPV infection was positively associated with CRC (OR 25.58; 95% CI 3.22 to 203.49), *p* < 0.001 (Table 2). The association between HPV-positive status and CRC was observed throughout the anatomical regions of the colon.

### 3.3. Sociodemographic, Lifestyle, and Clinicopathological Characteristics of HPV-Positive Cases

The mean age of the HPV-positive CRC cases was 60.3 years (ranging from 45 to 86; 9 were males). No significant associations were found between HPV-positive status and the following: gender, age, use of tobacco or alcohol, history of diabetes, family history of any cancer, or family history of CRC (Table 3). No significant associations were observed between HPV status and histological differentiation, tumor stage, or location (Table 4).

### 3.4. HPV-16 Genotyping and Assessment of Viral Physical Status

HPV-16 was detected in 12 of the 19 HPV-positive CRC samples (63.2%); the remaining 7 (36.8%) corresponded to other HPV genotypes (nontyped). HPV-16 was not detected in the HPV-positive control sample. The distributions of HPV-16 positive tumors regarding tumor location were as follows: 33.3% (4 of 12) of the samples were found in the proximal colon (cecum, ascending colon, and transverse colon), 50.0% (6 of 12) were found in the distal colon (splenic flexure, descending colon, and sigmoid colon), and 16.7% (2 of 12) were detected in the rectum. For non-HPV-16 tumors, 2 out of 7 (28.6%) were located in the distal colon and out 5 of 7 (71.43%) were found in the rectum. The physical status of HPV-16 DNA in CRC tissues was determined by detection of HPV-16 E2. The amplicon corresponding to the intact E2 was not detected in any of the HPV-16 positive cases indicating that the HPV-16 genome was integrated.

## 4. Discussion and Conclusions

HPV has been detected in colorectal tumors [13, 30–33]. However, the role of HPV in colorectal carcinogenesis has not been elucidated and the subject remains controversial. In the current investigation, we report a rigorous evaluation of HPV infections in colorectal tissues (CRC cases and normal mucosa controls) from a well-characterized Hispanic population reported to have a high incidence of HPV-associated malignancies [21–24] and a high CRC mortality rate [20]. HPV-16 genotyping and the physical status of the HPV-16 genome were also assessed.

A high prevalence of HPV infection (42.2%) was detected in the tumor tissues analyzed. Only 1 of the 36 (2.8%) control samples was positive for a non-HPV-16 type. The prevalence of HPV infections in colorectal tumors found in our cohort is comparable to percentages previously reported in studies from different countries using different experimental approaches [34]. Immunohistochemical analysis of formalin fixed paraffin-embedded (FFPE) tissue samples from individuals in the USA (*n* = 103; 30 controls, 30 adenomas, and 43 CRC) detected HPV DNA in 27% and 69% of the adenomas and CRC, respectively [35]. HPV type-specific PCR analyses of DNA from FFPE samples from Chinese individuals detected HPV in 29% of the adenomas (*n* = 37) and in 53% of the CRC (*n* = 70) [36]. Using the same nested PCR strategy used in this study in combination with in situ PCR, another study detected HPV DNA in 51% of the CRC samples (*n* = 55) and not in control samples (*n* = 10) from individuals in the USA [13]. Other studies using the same nested PCR strategy with CRC samples from Argentinean individuals detected HPV in 44% of CRC samples (*n* = 75) [31]. However, Pérez et al. (2005) reported that HPV was present in 33% of the nonneoplastic colorectal tissues studied (*n* = 30) [37]. In contrast, several studies have failed to detect HPV and have suggested that HPV infection is not a risk factor for CRC [38–41].

HPV-16 is the most common high-risk type in the Americas [30, 42]. HPV-16 has also been reported to be highly prevalent in HPV-associated malignancies in PR such as head and neck [43] and cervical cancer [44]. In our study, HPV-16 was the most prevalent high-risk HPV type detected; HPV-16 was present in 63.2% of the HPV-positive CRC samples (12 of 19), which is comparable to some previous reports where HPV-16 was the most prevalent high-risk HPV type detected. Similarly, analysis of HPV-positive CRC samples using a PCR-based HPV-16 genotyping method found that it was the most frequently detected viral type, being present in 41 of the 60 cases (68.3%) [14]. HPV-16 genotyping using the same primer pairs used in this study detected HPV-16 in 36% of the CRC samples (*n* = 107) [13]. Using non-PCR-based HPV-16 genotyping techniques, HPV-16 has been found in 16–94% of HPV-positive CRC samples [37, 45, 46].

The discrepancies in the literature reporting the detection of HPV and HPV-16 in colorectal tumors can be attributed to methodological differences including study design, type of tissue sample, sample size, sample collection, and differences in the sensitivity of the HPV detection or genotyping technique used. Frozen tissue samples were used because the DNA extracted is of superior quality compared to DNA from FFPE, which is degraded [47, 48]. The fixation procedure degrades DNA to fragments <200 bp, which results in decreased PCR yields and the inability to amplify long targets [49]. Therefore, DNA extracted from FFPE is not optimal for the nested PCR strategy used in our analyses. The PGMY/GP+ primer set combination amplifies HPV* L1* (450 bp). This primer combination has been found to be more type sensitive than the MY/GP+ primer sets, detecting a wider range of HPV types [50]. Additional differences in the detection of HPV in CRC have been associated with regional variations in the prevalence of HPV infection, which are influenced by the subject's racial/ethnic and geographical background [30, 34]. For example, HPV-18 is most frequently detected in CRC cases from Asia and Europe [30].

HPV was detected in CRC tissues in multiple tumor samples obtained throughout the colon. HPV was detected in 21.05% of proximal tumors, 42.11% of distal tumors, and 36.84% of the tumors in the rectum. The wide anatomical distribution of HPV infections in CRC tumor tissue implies that HPV infection in CRC is not a result of a retrograde viral transmission from the anogenital area. Given these results, we cannot discard the possibility of hematologic spread, as suggested by Bodaghi et al. [29]. There is evidence of HPV infections in infants and female university students who have never had penetrative sex, supporting that HPV transmission via other nonsexual routes may exist [51, 52]. Although HPV is a sexually transmitted disease, we did not collect sexual behavior information, which would have allowed us to assess whether there is any association between CRC and sexual behavior.

Virus integration into the host genome is a critical step in cervical carcinogenesis. Integration of HPV in the host genome has been reported in approximately 90% of cervical carcinomas. These cases show expression of the E6 and E7 viral oncogenes [53]. The HPV physical status can be detected by the absence of a PCR product since the E2 open reading frame is disrupted when HPV integrates into the host genome [54]. Potential limitations using this method could include the following: the assay can only detect integrated viral DNA in the absence of episomal HPV DNA, it cannot discriminate between pure episomal and mixed forms, and the possibility of viral integration without losing the E2 gene fragment is not considered [55, 56]. However, in our study, all of the HPV-16 positive cases tested showed integration, which discards the presence of episomal genomes. The high percentage of HPV-16 genome integration into the host genome supports the possibility that HPV may have a role in colorectal carcinogenesis. Our results, although encouraging, need to be interpreted cautiously. Despite the high prevalence of infection in colorectal tumors with a high-risk HPV type (HPV-16) and evidence of viral genome integration, future studies need to assess if the HPV infections are active and if the oncoproteins E6 and E7 are being expressed in order to contribute and/or have a causal role in colorectal carcinogenesis.

Our study has a relatively small sample size, which can be a limitation for stratified and restricted analyses. These could result in some imprecise estimates (as evidenced by the wide 95% confidence intervals). Nevertheless, the magnitude of the observed odds ratios for the association between HPV and CRC is very high and unlikely to result from a type I statistical error. The data generated in this study in combination with reports in the literature are not yet sufficient to conclude that HPV is a causative agent of CRC according to Bradford Hill's Criteria of Causation [57]. Five of the 9 postulated criteria are met. (1) Strength: strong statistically significant associations have been reported in this study and in the literature [30]. (2) Consistency: HPV has been detected in CRC in other studies using the same methodology [13, 31, 37]. (3) Plausibility: HPV integrates into the host genome and expresses oncogenic proteins known to promote carcinogenesis [16, 17]. (4) Analogy: high-risk HPVs, such as HPV-16, have a causal role in other cancers [6–9] such as cervical cancer [3–5]. (5) Temporality: HPV has also been detected in adenomas, CRC precursor lesions [35, 36]. Additional longitudinal studies evaluating the role of HPV during colorectal carcinogenesis are needed to fully support the consistency and temporality criterion. In conclusion, this study reports a high prevalence of HPV infection, a high prevalence of HPV-16 (a high-risk type), and integration of the HPV-16 genome in colorectal tumor tissue from Caribbean Hispanics. Further analyses are warranted in order to establish a causal association between HPV and CRC.

## Figures and Tables

**Table 1:** Summary of demographic and clinical history characteristics of the study participants.

Characteristics		CRC cases *n* = 45 *n* (%)	Controls *n* = 36 *n* (%)	*p* value
Gender				
Male		24 (53.3)	15 (41.7)	0.30
Female		21 (46.7)	21 (58.3)	Reference
Age median		61 (38–86)	60 (42–85)	
≥61		23 (51.1)	18 (50)	0.92
<61		22 (48.9)	18 (50)	Reference
Lifestyle				
Ever smoked cigarettes	Yes	24 (53.3)	14 (38.9)	0.20
	No	21 (46.7)	22 (61.1)	Reference
Ever drunk alcohol	Yes	25 (55.6)	15 (41.7)	0.21
	No	20 (44.4)	21 (58.3)	Reference
Clinical history				
Diabetes diagnosis	Yes	11 (25.6)	6 (17.1)	0.37
	No	32 (74.4)	29 (82.9)	Reference
Family history of any cancer	Yes	32 (71.1)	29 (82.9)	0.22
	No	13 (28.9)	6 (17.1)	Reference
Family history of CRC	Yes	8 (17.8)	15 (42.8)	0.01
	No	37 (82.2)	20 (57.1)	Reference

The number of cases may vary between categories according to the availability of the information. *p* values were calculated using chi-square tests or Fisher's exact tests, when appropriate.

**Table 2:** Association between HPV infection status and CRC.

Characteristics	CRC cases *n* = 45 *n* (%)	Controls *n* = 36 *n* (%)	*p* value	OR (95% CI)
HPV infection status				
HPV (+)	19 (42.2)	1 (2.8)	*p* < 0.001	25.58 (3.22–203.49)
HPV (−)	26 (57.8)	35 (97.2)		Reference
HPV infection status per colorectal subsite				
Proximal				
HPV (+)	4 (8.9)	1 (2.8)	0.001^*∗∗*^	46.67 (3.71–1175.05)^*∗*^
HPV (−)	3 (6.7)	35 (97.2)		Reference
Distal				
HPV (+)	8 (17.8)	1 (2.8)	*p* < 0.001^*∗∗*^	25.46 (3.32–582.87)^*∗*^
HPV (−)	11 (24.5)	35 (97.2)		Reference
Rectum				
HPV (+)	7 (15.6)	1 (2.8)	0.002^*∗∗*^	20.42 (2.62–474.77)^*∗*^
HPV (−)	12 (26.7)	35 (97.2)		Reference

*p* values were calculated using chi-square tests or Fisher's exact tests, when appropriate. ^*∗*^Mid-*p* exact method; ^*∗∗*^Fisher's exact test.

**Table 3:** Summary of the demographic, lifestyle, and clinical characteristics of the CRC cases according to HPV status.

		HPV (+) CRC *n* = 19 *n* (%)	HPV (−) CRC *n* = 26 *n* (%)	*p* value	OR (95% CI)
Gender					
Male		9 (47.4)	15 (57.7)	0.49	0.66 (0.20–2.17)
Female		10 (52.6)	11 (42.3)		Reference
Age median		59 (45–86)	61.5 (38–83)		
≥61		8 (42.1)	15 (57.7)	0.30	0.53 (0.16–1.77)
<61		11 (57.9)	11 (42.3)		Reference
Lifestyle					
Ever smoked cigarettes	Yes	9 (47.4)	15 (57.7)	0.49	0.66 (0.20–2.17)
	No	10 (52.6)	11 (42.3)		Reference
Ever drunk alcohol	Yes	9 (47.4)	16 (61.5)	0.35	0.56 (0.17–1.86)
	No	10 (52.6)	10 (38.5)		Reference
Clinical history					
Diabetes diagnosis	Yes	4 (21.1)	7 (29.1)	0.73^*∗∗*^	0.65 (0.14–2.73)^*∗*^
	No	15 (78.9)	17 (70.8)		Reference
Family history of any cancer	Yes	16 (84.2)	16 (61.5)	0.10	3.33 (0.77–14.42)
	No	3 (15.8)	10 (38.5)		Reference
Family history of CRC	Yes	3 (15.8)	5 (19.2)	*p* > 0.99^*∗∗*^	0.79 (0.14–3.95)^*∗*^
	No	16 (84.2)	21 (80.8)		Reference

The number of cases may vary between categories according to the availability of the information.

*p* values were calculated using chi-square tests or Fisher's exact tests, when appropriate. ^*∗*^Mid-*p* exact method; ^*∗∗*^Fisher's exact test.

**Table 4:** Clinicopathological characteristics of colorectal tumors according to HPV status.

	HPV (+) CRC *n* = 19 *n* (%)	HPV (−) CRC *n* = 26 *n* (%)	*p* value	OR (95% CI)
Differentiation				
Poor	1 (5.3)	1 (3.8)	*p* > 0.99^*∗∗*^	1.14 (0.03–47.21)^*∗*^
Well/moderate	14 (73.7)	16 (61.5)		Reference
Staging				
Advanced (III and IV)	7 (38.9)	6 (37.5)	0.93	1.06 (0.27–4.24)
Early (0, I, and II)	11 (61.1)	10 (62.5)		Reference
Tumor location				
Proximal	4 (21.1)	3 (11.53)	0.43^*∗∗*^	2.04 (0.37–12.16)^*∗*^
Distal and rectum	15 (79.0)	23 (88.5)		Reference

The number of cases may vary between categories according to the availability of the information. *p* values were calculated using chi-square tests or Fisher's exact tests, when appropriate. ^*∗*^Mid-*p* exact method; ^*∗∗*^Fisher's exact test.
